# Ultrasonographic assessment of abdominal aortic elasticity in hypertensive dogs

**DOI:** 10.1111/jvim.15891

**Published:** 2020-09-19

**Authors:** Andrea Corda, Francesca Corda, Domenico Caivano, Laura Saderi, Giovanni Sotgiu, Alessandra Mollica, Francesco Birettoni, Francesco Porciello, Maria Luisa Pinna Parpaglia

**Affiliations:** ^1^ Department of Veterinary Medicine Veterinary Teaching Hospital, University of Sassari Sassari Italy; ^2^ Department of Veterinary Medicine Veterinary Teaching Hospital, University of Perugia Perugia Italy; ^3^ Department of Biomedical Sciences University of Sassari Sassari Italy

**Keywords:** arterial stiffness, blood pressure dog, canine systemic hypertension, vascular ultrasonography

## Abstract

**Background:**

Systemic hypertension (SH) is a persistent and pathological increase in arterial blood pressure (BP). Chronic SH leads to an increase in aortic (Ao) stiffness, and measuring Ao elasticity is useful for estimating Ao stiffness in humans. Currently, no literature in veterinary medicine describes noninvasive assessment of abdominal Ao elasticity in dogs with SH.

**Objective:**

Compare ultrasonographic‐derived abdominal Ao strain (AoSt) between hypertensive (HT) and normotensive (NT) dogs.

**Animals:**

Fifty privately‐owned dogs with clinical signs, conditions, or both potentially associated with SH.

**Methods:**

Prospective observational case‐control study. Aortic stiffness was estimated by calculating AoSt as follows: AoSt = ([AoDs − AoDd]/AoDd) × 100, where AoDs and AoDd are the Ao diameter in systole and in diastole, respectively. Aortic stiffness was calculated from 2 different Ao transverse sections, the first caudal to the left renal artery (K_AoSt), and the second cranial to the external iliac arteries (I_AoSt).

**Results:**

Thirty‐two dogs were included in the HT group and 18 in the NT group. Both K_AoSt and I_AoSt in HT dogs were significantly lower (*P* < .05) than in NT dogs (7.4 ± SD 3.6) vs 10.3 (±3.8) and 5.7 (interquartile range [IQR], 3.9‐7.5) vs 8.1 (IQR, 7‐10.3), respectively. Only K_AoSt was significantly influenced by age.

**Conclusions and Clinical Importance:**

Ultrasonographic Ao elasticity assessment was feasible to compare HT and NT dogs. Results indicated that K_AoSt and I_AoSt indices can be used to assess SH‐related Ao stiffness, especially when indirect BP measurements are inconsistent or inaccurate. Additional studies to assess the AoSt in healthy dogs of various ages are needed.

**Abbreviations**
AKI: acute kidney injury
Ao: aorta
AoDd: aortic diameter in diastole
AoDs: aortic diameter in systole
AoSt: aortic strain
BP: blood pressure
BW: body weight
CI: confidence intervals
CKD: chronic kidney disease
DM: diabetes mellitus
HAC: hyperadrenocorticism
HDO: high definition oscillometry
HR: heart rate
HT: hypertensive dogs
I: aortic transverse section at iliac level
I_AoSt: aortic strain measured at iliac level
ICC: interclass coefficient correlation
IQR: interquartile range
K: aortic transverse section at renal level
K_AoSt: aortic strain measured at renal level
KD: kidney disease
NT: normotensive dogs
OPBA: Ethical Committee of the University of Sassari
PH: pulmonary hypertension
SBP: systolic blood pressure
SH: systemic hypertension
TOD: target organ damage
TSH: thyroid‐stimulating hormone

## 1 INTRODUCTION

Systemic hypertension (SH) is a persistent and pathological increase in arterial blood pressure (BP). Most commonly, secondary SH is diagnosed in dogs and cats as a consequence of various diseases, including renal diseases and endocrine disorders.^1^ Systemic hypertension, especially when sustained high BP occurs, can lead to severe target organ damage (TOD). Affected target organs include the kidneys, eyes, central nervous system, heart and vessels.^1^ Early and accurate diagnosis of SH is essential to minimize TOD. In clinical practice, Doppler and oscillometric devices are commonly used to noninvasively and indirectly estimate BP.^1^ However, excessive movements or tremors can make it difficult to measure BP using such devices. Moreover, anxiety or excitement can induce situational SH in dogs and cats, leading to an erroneous diagnosis of SH.^1^

The aorta (Ao) is the main distributing artery of the animal body. It dampens the pressure pulsations generated by intermittent left ventricular ejection, and transforms the pulsatile flow into continuous blood flow. During systole, the volume of blood ejected into the Ao dilates the vessel and promotes the storage of elastic energy within its walls. This energy then is returned during diastolic recoil, which forces blood into the circulatory system.

The dampening function is a consequence of the elastic properties of the Ao walls that allow it to dilate during systole and recoil during diastole.^2^ The elastic properties of the Ao are determined mainly by the greater proportion of elastin fibers versus smooth muscle and collagen contained in its walls.^2^, ^3^ In both humans and animals, chronic SH causes structural alterations in the arterial walls, characterized by smooth muscle cell hypertrophy and an increase in collagen.^4^, ^5^, ^6^, ^7^ This pathological remodeling results in an increase in Ao wall thickness and stiffness.^8^, ^9^, ^10^, ^11^ As Ao stiffness increases, Ao elasticity decreases. Several noninvasive diagnostic methods have been used to measure Ao elasticity in human medicine.^11^, ^12^, ^13^, ^14^, ^15^, ^16^, ^17^, ^18^, ^19^, ^20^, ^21^

In small animal practice, noninvasive measurement of Ao elastic properties could help clinicians differentiate between secondary or idiopathic SH and situational SH, as well as to overcome measurement difficulties caused by animal movements and tremors.

Our main objective was to assess abdominal Ao elasticity using ultrasonographic‐derived Ao strain (AoSt) in hypertensive (HT) and normotensive (NT) dogs. We also evaluated the effect of age, sex, reproductive status, body weight (BW), and heart rate (HR) on AoSt.

## 2 MATERIALS AND METHODS

The study was an observational case‐control study carried out at the Veterinary Teaching Hospital of the University of Sassari. The local Ethical Committee of the University of Sassari (OPBA) approved the study protocol, and all owners signed an informed consent form before enrollment of their animals. Dogs with clinical signs or conditions potentially associated with SH were prospectively included in the study.

The following diseases or conditions were considered potentially associated with SH: chronic kidney disease (CKD), acute kidney injury (AKI), spontaneous hyperadrenocorticism (HAC), diabetes mellitus (DM), hypothyroidism, ultrasonographic evidence of adrenal neoplasia, and glaucoma.^1^ The following clinical signs were considered potentially associated with SH: acute onset of blindness, hyphema,^1^, ^22^, ^23^, ^24^ and epistaxis^25^ as well as intracranial neurological signs characterized by acute or hyperacute onset, suggestive of cerebrovascular diseases, such as seizures, altered mentation, altered behavior, disorientation, ataxia, head tilt, and nystagmus.^1^, ^24^, ^26^, ^27^ Traumatized dogs and dogs receiving antihypertensive agents such as angiotensin‐converting enzyme inhibitors, angiotensin receptor blockers, calcium channel blockers, α_1_‐blockers, hydralazine, spironolactone, 𝛽‐blockers, thiazide and loop diuretics were excluded from the study. Dogs under treatment with drugs known to induce secondary SH (eg, glucocorticoids, phenylpropanolamine, toracenib phosphate) and dogs receiving anesthetic, sedative or opioid drugs during the 12 hours before starting the study procedures also were excluded. Diagnosis of CKD, AKI, spontaneous HAC, DM and hypothyroidism was based on a combination of anamnestic, clinical, laboratory, and ultrasonographic variables consistent with these diseases. Azotemia was defined as serum creatinine concentration ≥1.8 mg/dL (upper laboratory reference value).

A diagnosis of CKD was made based on the presence of clinical signs consistent with CKD (polyuria and polydipsia [PU/PD], chronic hyporexia, chronic weight loss, or a combination of these) associated with at least 1 of the following: (1) azotemia and ultrasonographic signs of CKD; (2) azotemia and inappropriate urine concentration (urine specific gravity [USG] <1.025); (3) absence of azotemia, USG <1.025 and ultrasonographic signs of CKD, in the absence of other causes of PU/PD; and (4) persistent proteinuria of renal origin.^28^

Proteinuria was defined as being persistent when a urinary protein‐to‐creatinine ratio of >0.5 was found repeatedly in ≥3 specimens obtained ≥2 weeks apart.^29^ Ultrasonographic changes consistent with CKD were increased echogenicity, irregular contour, decrease or absence of corticomedullary distinction, decreased kidney size, abnormalities in kidney shape or architecture, or a combination of these.^30^ A diagnosis of AKI was made if an acute onset of clinical signs attributable to AKI (anorexia, vomiting, diarrhea) was associated with ≥2 of the following criteria^31^: (1) presence of renal azotemia persisting at least 24 hours after correction of prerenal factors in a previously healthy dog; (2) ultrasonographic findings compatible with AKI, such as perirenal free fluid, and hyperechoic or enlarged kidneys or both; (3) increase in serum creatinine concentration >0.3 mg/dL or >25% from documented baseline during a 48‐hour interval in the absence of prerenal factors; (4) persistent pathological oliguria or anuria (<1 mL/kg/min) after volume repletion; and (5) evidence of acute renal tubular injury on urinalysis (renal glucosuria, urinary casts).

A diagnosis of spontaneous HAC was made based on the presence of clinical signs and laboratory findings suggestive of HAC associated with a positive result on a low‐dose dexamethasone suppression test.^32^ Diabetes mellitus was diagnosed by persistently marked hyperglycemia (plasma glucose concentration >250 mg/dL) and glucosuria in dogs with clinical signs consistent with the disease.^33^ A diagnosis of hypothyroidism was made based on the presence of clinical signs, CBC and serum biochemistry results suggestive of hypothyroidism associated with total T_4_ and free T_4_ concentrations below the reference range and canine serum TSH concentration above the reference range.^34^

All dogs in the study underwent complete physical examination, indirect BP measurement, and abdominal ultrasonography. Dogs with abnormal cardiac auscultatory findings (including murmur, arrhythmia, and gallop sounds) underwent complete echocardiographic examination with simultaneous electrocardiogram. We then excluded dogs with echocardiographic evidence of dilated cardiomyopathy,^35^ pericardial effusion, congenital cardiac disease, moderate‐to‐severe mitral valve regurgitation (regurgitant jet area/left atrium area ≥30%)^36^ and moderate‐to‐severe Ao valve regurgitation,^37^ with or without cardiac remodeling. Dogs with persistent arrhythmia and with Doppler echocardiography‐derived evidence of pulmonary hypertension (PH), defined as the presence of a tricuspid or pulmonic valve or both regurgitant jet velocity ≥2.8 and ≥2.2 m/s respectively,^38^ also were excluded.

Blood pressure was measured by an experienced operator (Francesca Corda). Measurements were performed in a quiet room, away from other animals, in the presence of the owner, after an acclimation period of 5 to 10 minutes. Dogs were placed in right lateral recumbency on a clinical examination table or on the floor, and kept in place by minimal restraint.

**FIGURE 1:**
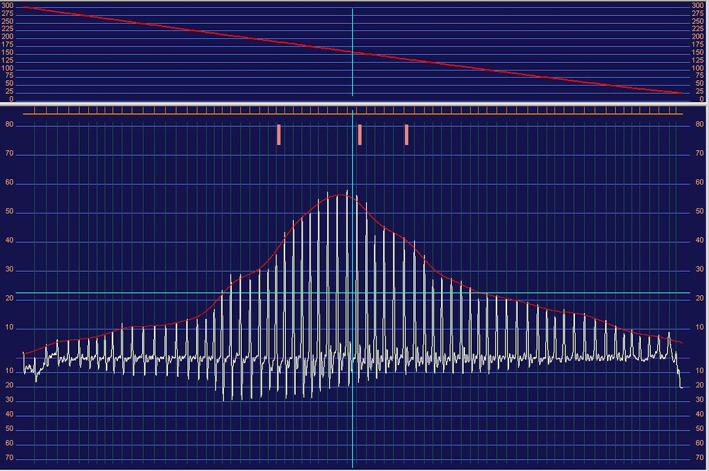
Example of an acceptable curve produced by the high definition oscillometric device in a hypertensive dog

All BP measurements were obtained using a high definition oscillometric (HDO) monitor (Memodiagnostic MD 15/90 Pro, S + B medVET, Germany) with a size‐specific cuff placed on the nonrecumbent cranial limb at the level of the heart. Cuff selection was based on the manufacturer's recommendation (C1 cuff for dogs weighing <10 kg, D1 cuff for dogs weighing 10‐25 kg, and D2 cuff for dogs weighing >25 kg). No measurement of circumference of the cuff application site was necessary, because HDO detects cuff volume and information on arterial diameter during the first reading, leading to the automatic adjustment of relevant parameters. During each session, several consecutive measurements of systolic, mean, and diastolic BP were obtained over a 5‐10‐minute period. The quality of measurements was assessed visually, in real time, on a laptop screen connected to the HDO device. The first measurement was discarded and only measurements with a normal pulse wave distribution were considered of good quality (Figure 1).

Systolic blood pressure (SBP) results, obtained from 5 to 7 consecutive measurements of good quality, that did not vary >10 mm Hg from each another, were averaged, and the mean result was recorded. Dogs were considered HT if SBP ≥160 mm Hg was obtained on at least 3 consecutive occasions within 2 weeks. Only HT dogs with neurological or ocular signs suggestive of TOD received a single BP measurement before inclusion in the study because they immediately were started on antihypertensive treatment.

**FIGURE 2:**
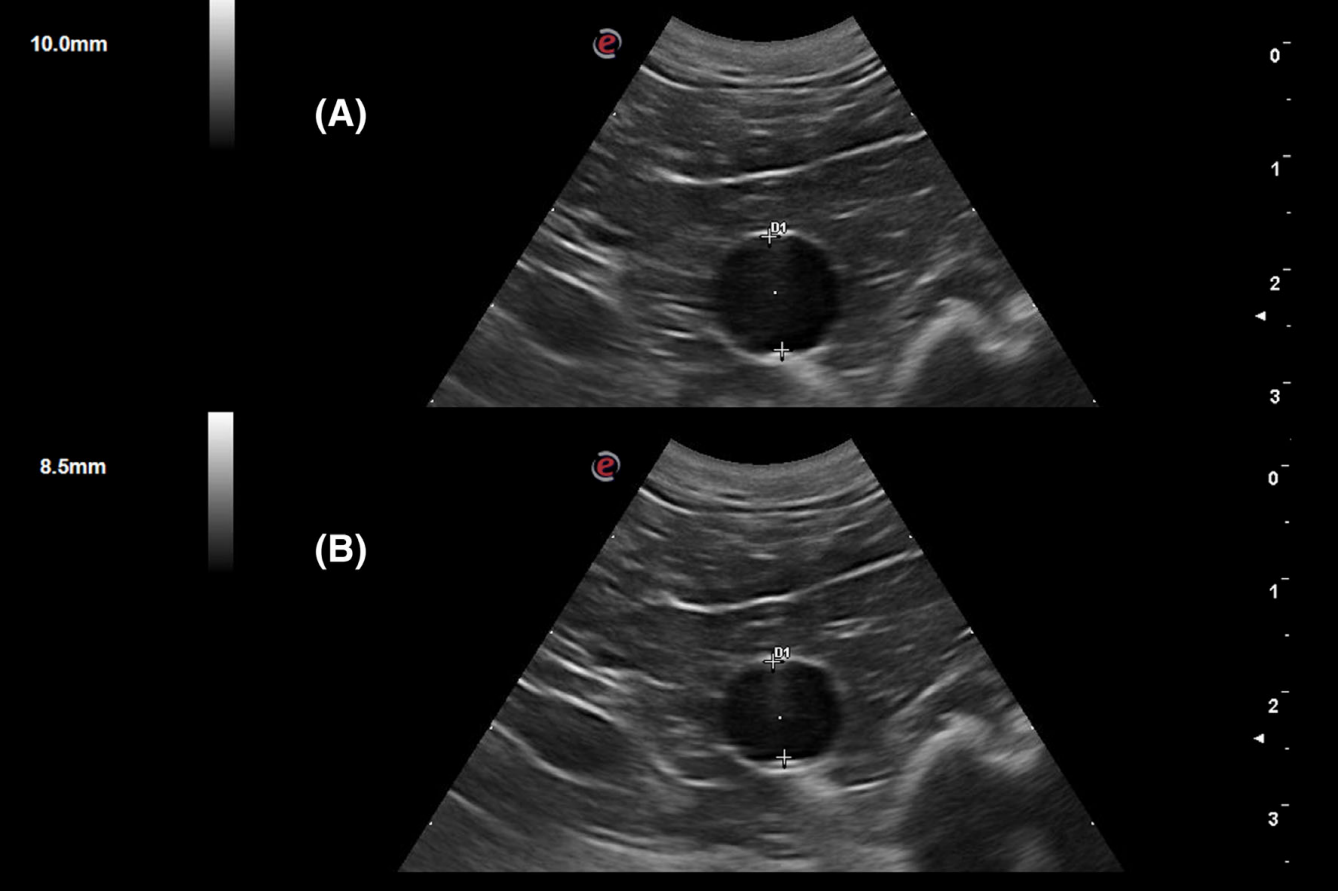
Transverse section of the abdominal aorta cranially to the emergence of the external iliac arteries of a normotensive patient; A, systolic diameter; B, diastolic diameter

Abdominal ultrasonographic examinations were performed by an individual experienced operator (Andrea Corda), within 12 hours of the last BP measurement, using a portable ultrasound unit (My Lab Alpha, Esaote, Florence, Italy) equipped with a multifrequency (4‐9 MHz) microconvex transducer (SC3123, Esaote, Florence, Italy). Dogs were positioned in right lateral recumbency on a clinical examination table. Transverse sections of the abdominal Ao were obtained by placing the probe in the left dorsal plane, with the ultrasound beam as perpendicular as possible (orthogonal) to the long axis of the vessel. Several 10‐second B‐mode cineloops of transverse Ao sections were acquired at 2 different levels: the first cranial to the origin of the external iliac arteries (I), between the origin of the external iliac arteries and the deep circumflex arteries, and the second caudal to the origin of the left renal artery (K). Offline measurements of Ao diameters were performed manually by a third operator (Domenico Caivano) unaware of the clinical history of the dogs and of the BP measurement results, from still B‐mode images after reviewing cineloop frames, with the inner edge‐to‐inner edge method^39^ (Figure 2).

Only transverse images of the abdominal Ao were measured, in which the vessel appeared circular and the arterial wall was clearly distinguishable. Five consecutive measurements of the latero‐lateral maximal systolic (AoDs) and minimal diastolic (AoDd) Ao diameters were averaged, and the mean values recorded. Abdominal Ao elasticity was estimated by calculating the AoSt, which is the percentage change in Ao diameter, obtained by the following formula: AoSt = ([AoDs−AoDd]/AoDd) × 100.^11^, ^13^, ^15^, ^21^

Aortic St was calculated at both anatomical levels, I and K, so as to obtain I_AoSt and K_AoSt, respectively. To determine intraobserver I_AoSt and K_AoSt measurement variability, 10 ultrasonographic examinations of 10 different dogs (5 from the HT group and 5 from the NT group) were randomly selected using the Microsoft Excel (Microsoft corporation, Redmond, Washington) random generation function and submitted to 3 repeated measurements by the same observer (Domenico Caivano). To assess intraobserver within‐day and between‐day variability, Ao diameters were measured twice in a single day and once at an interval of 1 week, respectively.

### 2.1 Statistical analysis

An ad hoc electronic form was used to collect all study variables. Qualitative variables were described using absolute and relative frequencies, whereas quantitative variables were summarized by means and SDs or medians and interquartile range (IQR) for parametric and nonparametric distributions, respectively. Chi‐squared or Fisher's exact tests were used to detect any statistical differences in the comparison of qualitative variables between HT and NT dogs. In‐between group comparisons of quantitative variables were performed using the Student *t* tests and the Mann‐Whitney test for parametric and nonparametric variables, respectively. Linear regression analyses were carried out to assess the relationship between sex, reproductive state, age, BW, HR, and AoSt.

Within‐day and between‐day intraobserver I_AoSt and K_AoSt measurement variability was assessed using the interclass coefficient correlation (ICC), which was reported with 95% confidence intervals (CI), and interpreted as follows: ICC < .5 is indicative of poor reliability, ICC values between .5 and .75 indicate moderate reliability, values between .75 and .9 indicate good reliability, and values >.9 indicate excellent reliability.^40^ A 2‐tailed *P* value <.05 was considered statistically significant. The programs STATA v. 14 (StatsCorp, TX) and MedCalc Statistical Software v. 18.11.3 (MedCalc Software bvba, Ostend, Belgium) were used to perform statistical computations.

## 3 RESULTS

Sixty‐one dogs initially were enrolled in the study, but 11 were excluded because of echocardiographic evidence of moderate‐to‐severe mitral regurgitation (n = 8), aortic regurgitation (n = 2), and PH (n = 1). Finally, 50 dogs met the inclusion criteria. Most of the dogs recruited for the study suffered from kidney disease (KD; n = 43; 86%), of which 41 (82%) were affected by CKD and 2 (4%) by AKI. One dog suffered from HAC (2%), 1 from DM (2%), and 5 (10%) showed neurological clinical signs potentially associated with SH.

Owner‐reported clinical signs were PU/PD (n = 26), weight loss (n = 23), decreased appetite (n = 15), lethargy (n = 9), anorexia (n = 7), poor hair coat (n = 6), tremors (n = 6), vomiting (n = 4), acute blindness (n = 4), ataxia (n = 3), abdominal distension (n = 3), diarrhea (n = 3), panting (n = 3), cough (n = 3), impaired exercise tolerance (n = 2), polyphagia (n = 2), anuria (n = 2), change in mentation (n = 2), nystagmus (n = 1), seizures (n = 1), impaired vision (n = 1), alopecia (n = 1), head tilt (n = 1), circling (n = 1), and falling (n = 1).

Thirty‐two dogs were included in the HT group (BP ≥160 mm Hg), and 18 in the NT group (BP ≤159 mm Hg). Dogs included in the HT group were crossbred (n = 19), Labrador Retriever (n = 3), English Setter (n = 1), Fonni's Dog (n = 1), Argentine Dogo (n = 1), Pointer (n = 1), Maremma Sheepdog (n = 1), Cane Corso (n = 1), German Shepherd (n = 1), Miniature Schnauzer (n = 1), Epagneul Breton (n = 1), and Beagle (n = 1). Dogs included in the NT group were crossbred (n = 8), Yorkshire Terrier (n = 1), Dachshund (n = 1), Pinscher (n = 1), Epagneul Breton (n = 1), Fonni's Dog (n = 1), Jack Russell Terrier (n = 1), Deutsch Drathar (n = 1), Deutsch Kurzhaar (n = 1), Pointer (n = 1), and Cane Corso (n = 1).

Sex, reproductive state, age, BW, HR, and number of dogs suffering from KD and CKD were not significantly different between the 2 groups (Table 1). Hypertensive dogs had mean (SD) SBP of 192 (18.9) mm Hg. In the HT group, 29 dogs (90.6% of the HT dogs) were suffering from CKD, 20 of which (62.5% of the HT dogs) were affected by concomitant leishmaniosis, 1 by concomitant DM, and 1 by concomitant hypothyroidism. One dog was diagnosed with AKI, and 1 dog suffered from pituitary‐dependent HAC. Finally, 1 HT dog, presented for acute onset of ataxia, tremors and impaired vision, was classified as having idiopathic hypertension because an underlying primary disease was not diagnosed. Normotensive dogs had a mean (SD) SBP of 146.5 (11) mm Hg.

**TABLE 1:** Comparison of sex, reproductive status, age, body weight, heart rate, kidney disease, chronic kidney disease, I_AoSt % and K_AoSt % between hypertensive and normotensive dogs

Variables	HT (n = 32)	NT (n = 18)	*P* value
Males, n (%)	27 (84.4)	11 (61.1)	.06
Intact, n (%)	29 (90.6)	14 (77.8)	.23
Age, years, mean (SD)	7.7 (3.7)	9.3 (4.4)	.15
BW, Kg, median (IQR)	17.2 (8.2‐26.4)	11.2 (5.5‐26.3)	.26
HR, bpm, mean (SD)	107 (27.1)	103.4 (31)	.65
Kidney disease, n (%)	30 (93.7)	13 (72.2)	.08
CKD, n (%)	29 (90.6)	12 (66.7)	.06
I_AoSt %, median (IQR)	5.7 (3.9–7.5)	8.1 (7–10.3)	.001*
K_AoSt %, mean (SD)	7.4 (3.6)	10.3 (3.8)	.01*

Abbreviations: BW, body weight; CKD, chronic kidney disease; HR, heart rate; HT, hypertensive dogs; I_AoSt %, abdominal aortic strain measured cranially to the external iliac arteries emergence; IQR, interquartile range; K_AoSt %, abdominal aortic strain measured caudally to the left renal artery emergence; NT, normotensive dogs. *Significant *P* values (*P* < .05).

The diseases diagnosed in the NT group were CKD (n = 12; 66.7% of NT dogs), of which 4 had concurrent leishmaniosis, and 2 had concurrent HAC. One NT dog suffered from AKI, and 1 from DM. Four dogs in the NT group were included because of acute neurological signs potentially associated with SH.

In both groups, K_AoSt was higher than I_AoSt. The median (IQR) I_AoSt of HT dogs was significantly lower compared to NT dogs (5.7 [3.9‐7.5] vs 8.1 [7‐10.3]; *P* = .0009). The mean (SD) K_AoSt of HT dogs was significantly lower compared to NT dogs (7.4 [3.6] vs 10.3 [3.8]; *P* = .01; Table 1).

Linear regression analyses results indicated that the effect of sex, reproductive state, age, BW and HR on I_AoSt was not significant. On the other hand, K_AoSt was significantly affected by age (Table 2). Intraobserver within‐day and between‐day I_AoSt and K_AoSt measurement variability was considered moderate to excellent (Table 3).

**TABLE 2:** Effect of gender, age, reproductive status, body weight and heart rate on I_AoSt % and K_AoSt %

	Beta (95% CI)	*P* value
I_AoSt %		
Male	−2 (−4.1; 0.17)	.07
Age, years	−0.11 (−0.35; 0.13)	.35
Intact	−2.65 (−5.27; −0.04)	.05
BW, kg	−0.06 (−0.14; 0.02)	.12
HR, bpm	−0.03 (−0.06; 0.01)	.12
K_AoSt %		
Male	−0.61 (−3.21; 1.99)	.64
Age, years	−0.43 (−0.69; −0.18)	.001*
Intact	−1.01 (−4.20; 2.18)	.53
BW, kg	−0.08 (−0.17; 0.02)	.10
HR, bpm	−0.03 (−0.07; 0.01)	.12

Abbreviations: BW, body weight; CI, confidence intervals; HR, heart rate; I_AoSt %, abdominal aortic strain measured cranially to the external iliac arteries emergence; K_AoSt %, abdominal aortic strain measured caudally to the left renal artery emergence. *Significant *P* values (*P* < .05).

**TABLE 3:** Within‐day and between‐day intraoperator variability of I_AoSt % and K_AoSt %

	ICC	95% CI
Within‐day K_AoSt %	.94	0.75; 1
Within‐day I_AoSt %	.92	0.68; 0.98
Between‐day K_AoSt %	.91	0.73; 0.98
Between‐day I_AoSt %	.95	0.85; 0.99

Abbreviations: CI, confidence intervals; I_AoSt %, abdominal aortic strain measured cranially to the external iliac arteries emergence; ICC, interclass coefficient correlation; K_AoSt %, abdominal aortic strain measured caudally to the left renal artery emergence.

## 4 DISCUSSION

Our results indicate that Ao elasticity assessment, measured using ultrasonographically‐derived AoSt, was feasible in all dogs included in the study, with good intraobserver measurement variability. Aortic St indices measured at 2 different levels (I_AoSt and K_AoSt) were significantly lower in HT than in NT dogs, but only I_AoSt was not influenced by age. These preliminary results suggest that the I_AoSt index is useful when assessing dogs with SH, especially when errors in measurements, obtained using noninvasive indirect methods, are suspected.

Our findings are consistent with those published in human medicine. Several studies have shown an increase in Ao stiffness in humans suffering from chronic arterial hypertension.^9^, ^10^, ^11^, ^12^, ^20^, ^41^ In veterinary medicine, the echocardiographic‐derived right pulmonary artery distensibility index^42^, ^43^, ^44^, ^45^ has been used as an index of PH in dogs.^45^, ^46^

To the best of our knowledge, ours is the first study in which Ao stiffness has been assessed using ultrasonography in dogs with SH. Arteries subjected to a chronic increase in BP likely undergo remodeling of their walls, characterized by smooth muscle cell hypertrophy and increased collagen content, which then results in a decrease in vessel elasticity.^4^, ^5^, ^6^, ^7^

Although more advanced noninvasive methods are available for measuring Ao elasticity in humans,^10^, ^16^ ultrasonography is the most practical and readily available imaging technology for clinical use in dogs. Several ultrasonographic‐derived indices of Ao elasticity have been proposed to evaluate arterial stiffness.^16^, ^20^, ^21^, ^47^ Most of them include in their formula pulsatile pressure, which is the difference between systolic and diastolic BP.

We assessed Ao elasticity by measuring the percentage change in Ao diameter (AoSt) for 2 main reasons: first, in clinical practice measurement of BP by the Doppler method, which only indicates the systolic pressure, is more common than measurement by the oscillometric method; second, we sought an index that would complement BP measurement and therefore be independent of it. In addition, AoSt is a useful index of Ao elasticity in humans.^11^, ^15^, ^20^, ^21^, ^47^

In both groups, K_AoSt was higher than I_AoSt. The greater elasticity of the K‐level compared to the I‐level could be a result of the different composition of the Ao wall along its course. In fact, the concentration of elastic fibers gradually decreases from the proximal to the distal Ao, as the latter is subject to lower pulse pressure.^2^, ^3^, ^16^ The decrease in arterial elasticity is an alteration that also occurs with age^48^, ^49^, ^50^ and in CKD.^51^

In our study, these effects were minimized because no significant difference in age or CKD prevalence between the 2 groups was observed (Table 1). We evaluated the effect of age on I_AoSt and K_AoSt in all of our dogs (HT and NT), and K_AoSt was significantly affected by age, whereas I_AoSt was not. This difference could be because age‐related arterial stiffness is inversely related to distance from the heart.^3^, ^16^, ^49^, ^52^, ^53^ Given that the I‐level is more peripheral than the K‐level, it may be less affected by age‐related increases in Ao stiffness.

Most of our HT dogs suffered from KD (93.7%), consistent with previous studies.^54^ Many of the HT dogs (62.5%) were affected by concomitant CKD and leishmaniosis. Our results confirm the high prevalence of SH in dogs affected by leishmaniosis,^55^, ^56^ and emphasize the importance of the detection of BP in dogs affected by this infectious disease.

Our results suggest that ultrasonographic measurement of abdominal AoSt is highly repeatable when performed by a single experienced operator. Indeed, both I_AoSt and K_AoSt had intraobserver within‐day and between‐day measurement repeatability that was moderate to excellent. We were unable to find any previously reported variability data for ultrasonographic‐derived abdominal AoSt in dogs. In human beings, ultrasonographic measurements of abdominal Ao diameters seem to have low variability.^57^

The main limitation of our study was the absence of a control group consisting of healthy subjects of different ages. A control group would have allowed us, first, to establish normal values of I_AoSt and K_AoSt in healthy dogs, second, to study the effect of age on AoSt in the absence of pathology and, finally, to understand the difference of AoSt between healthy and pathological NT patients.

Another important limitation was the low number or absence of HT dogs affected by diseases other than CKD (eg, AKI, HAC, DM, hypothyroidism, pheochromocytoma), this limitation means that we cannot confirm that AoSt is decreased in all cases of SH.

In conclusion, our study showed that abdominal Ao elasticity, assessed by the ultrasonographically‐derived AoSt, was easy to obtain in dogs, and was significantly decreased in dogs affected by secondary SH. The AoSt could provide useful information in dogs with SH, especially when BP measurements obtained by noninvasive indirect methods are inconsistent or inaccurate.

## CONFLICT OF INTEREST DECLARATION

Authors declare no conflict of interest.

## OFF‐LABEL ANTIMICROBIAL DECLARATION

Authors declare no off‐label use of antimicrobials.

## INSTITUTIONAL ANIMAL CARE AND USE COMMITTEE (IACUC) OR OTHER APPROVAL DECLARATION

The study protocol was approved by the Ethical Committee of the University of Sassari (OPBA) protocol number 50675/18.

## HUMAN ETHICS APPROVAL DECLARATION

Authors declare human ethics approval was not needed for this study.
